# Women's Perspectives on the Unique Benefits and Challenges of Self‐Injectable Contraception: A Four‐Country In‐Depth Interview Study in Sub‐Saharan Africa

**DOI:** 10.1111/sifp.12277

**Published:** 2024-11-07

**Authors:** Emily Himes, Lauren Suchman, Martha Kamanga, Catherine Birabwa, Serah Gitome, Elizabeth Omoluabi, Sarah Okumu, Grace Nmadu, Zachary Kwena, Jenny Liu, Sneha Challa, Dinah Amongin, Pauline Wekesa, Louisa Ndunyu, Elizabeth Bukusi, Address Malata, Lynn Atuyambe, Mandayachepa Nyando, Chioma Okoli, Aminat Tijani, Janelli Vallin, Ayobambo Jegede, Shakede Dimowo, Alfred Maluwa, Phoebe Alitubeera, Betty Kaudha, Agnes Kayego, Tamandani Jumbe, Innocencia Mtalimanja, Peter Waiswa, Beth Phillips, Kelsey Holt

**Affiliations:** ^1^ Department of Family and Community Medicine University of California San Francisco San Francisco CA 94110 USA; ^2^ University of California San Francisco University of California San Francisco San Francisco CA 94158 USA; ^3^ Midwifery Department Kamuzu University of Health Sciences Blantyre 312225 Malawi; ^4^ School of Public Health Makerere University P. O. Box 7062 Kampala Uganda; ^5^ Centre for Microbiology Research Kenya Medical Research Institute Nairobi 00200 Kenya; ^6^ AkenaPlus Health Abuja 900108 Nigeria; ^7^ Department of Public Health Maseno University Maseno 40105 Kenya; ^8^ Departments of Global Health and Obstetrics and Gynecology University of Washington Seattle WA 98195 USA; ^9^ Department of Obstetrics, Gynecology and Reproductive Sciences University of California San Francisco San Francisco CA 94158 USA; ^10^ Academy of Medical Sciences Malawi University of Science and Technology Limbe 312229 Malawi; ^11^ Global Public Health Karolinska Institute Stockholm 171 77 Sweden; ^12^ Busoga Health Forum P.O.Box 805 Jinja Uganda

## Abstract

Implementing self‐injection (SI) of subcutaneous depot‐medroxyprogesterone acetate (DMPA‐SC) is a key self‐care strategy for sexual and reproductive health, but SI uptake remains low, and assertions about the potential of SI to increase women's control over contraceptive use lack evidence. We sought to qualitatively explore how women with diverse contraceptive experiences—including those with and without experience using SI—view the benefits and challenges of SI as compared to other methods. We conducted 241 in‐depth interviews with women across four sub‐Saharan African countries and found alignment between the perceived and experienced benefits of SI across our diverse sample. Through the benefits of privacy, easier access, and self‐management, we found SI can promote greater control over the contraceptive experience by facilitating a woman's ability to act on her preferences and control who is involved in or aware of her contraceptive use. Interviews revealed SI's potential is, however, constrained by inherent limitations in the method; for example, it is often not private or accessible *enough* and many fear injecting themselves. SI has the most potential when implemented with programmatic solutions that mitigate challenges women experience or anticipate and allow more women to benefit from the privacy, easier access, and self‐management that SI offers.

## INTRODUCTION

The World Health Organization (WHO) recommends integrating self‐injection (SI) of subcutaneous depot‐medroxyprogesterone acetate (DMPA‐SC) into the contraceptive method mix as a key strategy to promote self‐care in sexual and reproductive health (World Health Organization 2019). DMPA‐SC for SI offers contraceptive users the option to take home units to store for self‐administration after receiving training from qualified providers. Research across a number of settings has shown women can feasibly manage this process and generally find SI convenient, discreet, and easy (Family Planning 2030 2018; Burke, Packer, et al. 2018; Cover, Ba, et al. 2017; Hernandez et al. 2018; Jacinto et al. 2016; Cover, Lim, et al. 2017). Further, several studies report higher continuation rates among women self‐injecting with DMPA‐SC compared to those who use provider‐administered DMPA‐SC, suggesting that the option to self‐inject may enable more people to use contraception when they want to (Kennedy et al. 2019; Cover et al. 2018; Burke, Chen, et al. 2018). As DMPA‐SC has been progressively rolled out over the last decade, including in many sub‐Saharan African countries (Access Collaborative, n.d.‐b; n.d.‐c; Akinyemi et al. 2022; Wynne 2020), these continuation‐related results have been heralded as evidence that SI has “empowering” (Askew and Wells 2018; PATH, n.d.) potential. For example, authors of an opening editorial for a 2018 *Contraception* issue focused on DMPA‐SC asserted that “SI empowers women to have autonomy and control over their method of contraception”(Askew and Wells 2018). Yet, despite this frequently referenced promise of SI, the assertion that it offers something “empowering” (Askew and Wells 2018; PATH, n.d.) lacks evidence (Kennedy et al. 2019; Burke, Ridgeway, et al. 2022).

For those without reliable healthcare access or who face social stigma around sexual activity, family or partner disapproval of contraceptive use, or healthcare provider bias, SI does indeed offer the theoretical benefit of helping them circumvent these common barriers to contraceptive use (World Health Organization 2019; Chandra‐Mouli et al. 2014; Engelbert Bain, Amu, and Tarkang 2021; Chola, Hlongwana, and Ginindza 2023; Narasimhan et al. 2020; Blackstone, Nwaozuru, and Iwelunmor 2017). In particular, SI offers advantages over the highly popular DMPA delivered as an intramuscular injection (United Nations 2019) or provider‐administered DMPA‐SC because it allows for re‐injection without returning to a healthcare provider. However, low rates of SI use, even in countries where SI has been rolled out, suggest that many women either do not see value in the method or face barriers to using it. The most recent Performance Monitoring for Action data from 2021 show low SI use across sub‐Saharan Africa with the percentage of DMPA‐SC users who report self‐injecting ranging from 0 percent to 16.7 percent across sites and SI use declining in several sites since 2018 (Wood et al. 2022). Further, interest in SI among those not using contraception but considering an injectable method was low (1.5–11.4 percent) (Wood et al. 2022). These findings suggest limitations to the potential of SI that merit further investigation.

In the context of high enthusiasm for SI among donors and researchers coupled with low rates of real‐world use, more research is needed to understand women's perspectives on SI—including what they see, if anything, as uniquely beneficial compared to provider‐administered injectables and other methods, and what stands in their way of using it. In line with calls to better study how self‐care impacts autonomy, self‐efficacy, and empowerment (World Health Organization 2019; Burke, Ridgeway, et al. 2022), we sought to qualitatively explore the potential of SI from the perspective of reproductive‐aged women through interviews with women who had a range of contraceptive experiences (including SI users, users of other methods, and nonusers of contraception) in Kenya, Malawi, Nigeria, and Uganda.

## METHODS

The Innovations for Choice and Autonomy (ICAN) project aims to understand how SI of DMPA‐SC can be implemented in a way that best meets women's needs, as defined by women themselves, through a variety of research approaches—including qualitative research (the focus of this paper), human‐centered design, program evaluation, implementation science, and longitudinal cohort studies. ICAN research is conducted by a consortium of research partners in Kenya, Malawi, Nigeria, Uganda, and the United States. The present qualitative study, leveraging data from in‐depth interviews (IDIs), represents the formative phase of the ICAN project wherein we sought to empirically identify women's perspectives related to SI to inform implementation strategies in sub‐Saharan Africa. We took a modified grounded theory approach to this qualitative study in order to discover emergent patterns in how the option of SI fits into women's experience of contraceptive decision‐making, as described in Suchman et al. (2023).

### Ethical Approval

Ethical approval for the research was obtained from: the University of California San Francisco (UCSF) Institutional Review Board (270555, 270747, 270554, 270084); the Kenya Medical Research Institute's Scientific Ethics Review Unit (KEMRI/SERU/CMR/P00136/4013) and Kenyan National Commission for Science, Technology, and Innovation (464643); the Makerere University School of Public Health Research and Ethics Committee (812) and Uganda National Council of Science and Technology (HS1087ES); the Malawi University of Science and Technology Research Ethics Committee (P.03/2020/007); and the National Health Research Ethics Committee, Nigeria (01/01/2007‐25/09/2020).

### Study Sites, Recruitment, and Sampling

The dataset for this study consisted of 241 semistructured IDIs with women of reproductive age (ages 15–45) conducted from January to September 2021. Eight study sites were chosen based on where ICAN had been funded to design and evaluate SI service delivery approaches in the subsequent project phase. Study sites reflected a mix of urban and rural areas: Kisumu and Nairobi metropolitan areas (urban) in Kenya; Mulanje and Ntchisi, two rural districts in Malawi; Enugu and Plateau states, representing urban and rural areas of Nigeria; and the mostly rural districts of Mayuge and Oyam in Uganda.

We recruited participants directly in health services, at community events, and through client registers provided by public and private healthcare providers. In Kenya, Malawi, and Uganda, we received client registers from public sector community health workers and directly recruited women in clinics and at community outreach events. In Nigeria, we received client registers from public sector family planning supervisors and recruited in public sector facilities on immunization days. In Nigeria and Kenya, we also received referrals from private‐sector pharmacists and private‐sector patent and proprietary medicine vendors (Nigeria) known to sell DMPA‐SC.

Women were purposively sampled to ensure representation of two age groups (ages 15–19 years and ages 20–45 years) and diversity of contraceptive use experience (women who had ever used DMPA‐SC; ever used other forms of contraception, including “traditional” methods such as withdrawal or fertility awareness; and never used any form of contraception). A priori, we set 60 as the target number of interviews per country that would allow sufficient diversity in the sample by age, contraceptive use status, and geography based on team members’ prior experiences. As part of the rapid analysis process, we monitored emergent themes and continued interviews until reaching theoretic sufficiency (Suchman et al. 2023).

### Instrument

ICAN team members from each study country collaborated to develop the interview guide under the guidance of a qualitative advisory committee (QAC), which was comprised of qualitative researchers from each consortium partner and coordinated the process of developing study procedures and tools. The guide was developed to capture women's perspectives on SI (the focus of this paper) and internal and external factors influencing women's agency and autonomy in making and acting on contraceptive decisions (the focus of another forthcoming paper). Interview guide domains included (1) what women consider when making and acting on decisions regarding contraceptive use, including who in their lives influences these decisions; (2) women's perceptions of their right and the right of other women to make and act on their own contraceptive decisions; (3) previous experiences with and perspectives on contraceptive use; and (4) interest in and perspectives on SI. For participants who had not used SI, an explanation of the method and its administration was given, including specifics on training to self‐inject, how to access and use the method, and storage and disposal. Women who had used SI were asked additional questions regarding how they heard about the method, their training experience, access to the method, the act of self‐injecting, product disposal, and their reason for choosing SI. This paper focuses on data related to perspectives on SI, but, where relevant, also pulls from data regarding contraceptive decision‐making and previous experience with contraception. Native speakers from the research team translated the instrument and piloted it through a mix of in‐classroom practice among interviewers and field testing with women who met the study criteria. As part of the modified grounded theory approach to the study, the interview guide was further refined through collaboration across teams during a phase of early rapid analysis and reassessment of probing questions (Suchman et al. 2023).

### Data Collection Procedures

Women who agreed to participate in the study gave their written consent before being interviewed in either English or their preferred local language; in Malawi, a thumbprint was an alternative option to demonstrate consent, and in Nigeria, participants had the option for verbal consent. In Kenya, researchers conducted interviews in English, Dholuo, or Swahili; in Malawi, they conducted interviews in Chichewa; in Nigeria, they conducted interviews in English, Hausa, or Igbo; and in Uganda, they conducted interviews in Langi or Lusoga. Interviewers participated in a centralized consortium training developed by ICAN's QAC to refresh their interviewing skills, align on processes and techniques, and provide context on ICAN's research aims and guiding principles related to rights‐based family planning. All interviewers were bilingual in both English and a local language relevant to each study site and followed interview guides translated into the participant's preferred language. For the interview, participants decided on a private setting, frequently either at their house, a school compound, under a tree, or at an office in a health facility or research institution. In scenarios where the interviewer deemed the venue not conducive to privacy, they guided the respondent on an alternative to ensure privacy. Interviews lasted 1–2 hours and were audio‐recorded with the participant's informed consent. Participants received token monetary reimbursement at the conclusion of interviews to compensate for their time participating in the study.

### Analysis

Trained transcriptionists who were native speakers of the relevant local languages in each country and fluent in English simultaneously translated and transcribed interviews. A team of researchers from each study country coded the transcripts in Dedoose Version 9.0.17 (“Dedoose Version 9.0.17, Cloud Application for Managing, Analyzing, and Presenting Qualitative and Mixed Method Research Data (2022),” n.d.) using a codebook with both deductive codes representing major interview domains and inductive codes developed iteratively through a rapid analysis process (codebook development and coding process described elsewhere; Suchman et al. 2023).

For this analysis, two lead analysts (LS and EH) extracted relevant code reports on SI benefits and challenges (both experienced and perceived, depending on the participant's history of SI use), interest in SI, barriers to and facilitators of contraceptive use, and perspectives on covert contraceptive use (use without a male partner's knowledge), first by country and then by the following demographic categories: age (15‐19 and 20–45), residence (urban and rural), and employment (employed and unemployed). This stratified extraction of code output was done to assess how SI perspectives may differ across countries and sociodemographic groups. Further, a comparison of perspectives from participants with a range of contraceptive experiences facilitated the examination of whether the perceived benefits of SI from participants with no SI experience corresponded to actualized benefits in the lived experience of SI users in our sample. Lead analysts reviewed stratified code reports and drafted analytic memos related to the benefits and challenges of SI use (both perceived and experienced), organized by the aforementioned demographic categories.

Lead analysts then mapped out and discussed emergent themes, created summaries of major themes by demographic category and country, shared them with co‐authors for review, and received both written and verbal feedback. Lead analysts then finalized the results, drawing on co‐author feedback.

## RESULTS

### Participant Characteristics

Among our sample of 241 participants, 37 percent were aged 15–19 and 63 percent were aged 20–45; across both age groups, 37 percent had never used contraception (Table 1). Overall, 17 percent of participants had experience self‐injecting DMPA‐SC; in Kenya, no women were successfully recruited with SI experience. Most of the sample in each country had children and most were married, except in Kenya where 34 percent were married. Employment status varied by country, with the percentage of the sample employed ranging from 0 percent in Malawi (where the sample was primarily subsistence farmers) to 73 percent in Nigeria and Uganda. Participants in Nigeria and Kenya primarily lived in urban or peri‐urban areas (76 percent and 84 percent, respectively), whereas participants in Malawi lived in primarily rural (87 percent) areas, and in Uganda the sample was evenly mixed between urban/peri‐urban (47 percent) and rural (50 percent) participants.

**TABLE 1:** Participants’ demographic characteristics

Demographic variables		Kenya (*n* = 61)		Malawi (*n* = 60)		Nigeria (*n* = 60)		Uganda (*n* = 60)		Total (*N* = 241)	
Age		*n*	%	*n*	%	*n*	%	*n*	%	*N*	%
	15–19	25	41	18	30	16	27	29	48	88	37
	20–45	36	59	42	70	44	73	31	52	153	63
Contraceptive use history											
	Never used	25	41	22	37	20	33	23	38	90	37
	Prior or current use^a^	36	59	38	63	40	67	37	62	151	63
DMPA‐SC history											
	Never used	59	97	30	50	45	75	33	55	167	69
	Ever used DMPA‐SC	2	3	30	50	15	25	27	45	74	31
	Ever self‐injected DMPA‐SC	0	0	18	30	11	18	12	20	41	17
Parity^b^											
	Nulliparous	25	41	16	27	29	48	8	13	78	32
	Ever had children	35	57	43	72	31	52	45	75	154	64
Marital status^c^											
	Married	21	34	40	67	28	47	37	62	126	52
	Unmarried	40	66	19	32	32	53	11	18	102	42
	Partnered/cohabitating	0	0	0	0	0	0	5	8	5	2
	Married, the husband has multiple wives	0	0	1	2	0	0	3	5	4	2
Employment status^d^											
	Employed	22	36	0	0	44	73	44	73	110	46
	Unemployed	37	61	60	100^e^	16	27	14	23	127	53
Residence^f^											
	Rural	2	3	52	87	13	22	30	50	97	40
	Peri‐urban	40	66	8	13	11	18	16	27	75	31
	Urban	11	18	0	0	35	58	12	20	58	24

^a^ Contraceptive use was defined as using any effective method of pregnancy prevention, including so‐called “traditional” methods such as withdrawal or fertility awareness methods

^b^ Missing data from Kenya (*n* = 1), Malawi (*n* = 1), and Uganda (*n* = 7).

^c^ Missing data from Uganda (*n* = 4).

^d^ Missing data from Kenya (*n* = 2) and Uganda (*n* = 2).

^e^ Sample from Malawi consisted primarily of subsistence farmers who did not describe themselves as formally employed.

^f^ Missing data from Kenya (*n* = 8), Nigeria (*n* = 1), and Uganda (*n* = 2).

### Benefits of SI and the Limits of Those Benefits

Here we discuss three domains in which women perceive SI as uniquely beneficial: privacy, eased access barriers, and self‐management. These findings are based on both real experiences of SI users and perceptions of those not using SI on the role it could play in their lives. Within each domain, we describe women's perspectives on barriers to their ability to benefit from SI due to inherent limitations of the method or the way it is provided. Table 2 summarizes the identified benefits and limitations with supporting evidence from both SI users and those with no experience using SI.

**TABLE 2:** Illustrative data of perceived and realized benefits of SI with limitations of identified benefits

Domain	Description	Illustrative quotes from user groups	
		Women with no experience using SI	Women with experience using SI
*Privacy* benefits	Women can act on a desire to use contraception even when family members disagree	“If you don't have permission, then you can do it privately without anyone knowing.” *—*Nonuser of contraception in Uganda, aged 19	“I feel safe now because nobody knows what I do since I do it in the comfort of my house, but then someone you know can walk in when you are doing it in the facility.” —SI user in Nigeria (covert use), aged 24
	Women can keep contraceptive use a secret from community members	“The young girls who use family planning most of the time they are judged when they line up for family planning so if you have yours that you can use to inject yourself (in private) no one can judge you.” —Nonuser of contraception in Kenya, aged 17	“It gives the benefit of secrecy; no one gets to know what you are doing since you inject yourself in the privacy of your home.” *—*SI user in Uganda, aged 17
	SI helps women avoid exposing their bodies to a provider (Malawi only)^a^	“I liked the fact that it ensures privacy for the user. There is no body exposure to the other people.” *—*Nonuser of contraception in Malawi, aged 26	“I like it most because I self‐inject. Like for us who feel embarrassed exposing our bodies, we like it so much.” *—*SI user in Malawi, aged 23
Limitations	Privacy within the home is not a given: storage, disposal, or side effects may betray a woman's desire to keep use hidden from others	“There is nothing hard about it, I was just scared that my mother would have found me in the process of administering.” —DMPA‐SC user (provider administered) in Malawi, aged 17	“He may find (it) and say you have been denying but I have found it. That is why I leave it there at the VHTs then I go and pick it at once and inject myself.“ *—*SI user in Uganda (covert use), aged 27^b^
Benefits of *eased access barriers*	Women can access contraception without sacrificing time waiting in line or traveling to facilities	“I don't need to go to a health personnel to do it for me or to go to the hospital and start joining the queue, waiting for it to be administered [to me]. But, if I have it at home, every three months I can do it myself.” *—*DMPA‐IM and female condom user In Nigeria, aged 28	“It is so easy to inject yourself, and it also saves for me the time of going to the health facility, I now use that time to do other things.” *—*SI user in Malawi aged 32
	By storing units at home, women can be certain of the supply of contraception they have and how long it will last (a minor theme among SI users)^c^	“It's now at your disposal, it's now close to you. Maybe when you go to the health facility, they will tell you it's not in stock, that you have to go and come back. But with this one (SI), you know that at any time, you can use it.” —Nonuser of contraception in Nigeria, aged 25	“When you go home with the doses, you are safer, you don't risk going to the health center and you don't find the doses or the health workers.” *—*SI user in Uganda, aged 18
	Using SI at home means women avoid the financial burden of transport costs, provider visit fees, and reduced wages due to time away from work	“One of the benefits is that you will save money. Once you buy it and keep it to inject by yourself, nobody will ask you to pay before injection because some people when they want to inject you, they will demand for money.” *—*Nonuser of contraception in Nigeria, aged 30	“If you do it at home, you will not pay for anything. When you have it at home, it means you are free, no one will collect money from you, but if you go to the hospital, you must give something. And if the date of the injection comes, and you don't have money, then the date will pass.” — SI user from Nigeria, aged 34
	SI can be continued without interruption for women unable to travel to clinics (e.g., due to lack of transport, caring for a relative, COVID)	“What will make me use it is the responsibilities I have at home. I could be having a sick child at home and leaving my child alone to go get injection is tricky. It lessens the burden of walking to the health facility.” *—*DMPA‐IM user in Kenya, aged 19	“I manage to inject myself when it's my time to do so, while if I was going to the hospital may be some day I would not manage to go there because I wouldn't have transport, because there is a distance from my house to get to the hospital, failure to go to the hospital would result in pregnancy but because I have it in my house I cannot miss my dose.” *—*SI user in Malawi, aged 27
	Women can avoid anticipated provider bias, particularly towards young women (minor theme)^d^	“I will not go to that doctor because I'm afraid. How will she carry me? How will she take me? But when I'm given that method and I have the knowledge, then I'll be very much comfortable at home injecting myself.” —Oral contraceptive user in Kenya, aged 17	“They will ask so many questions (at the health facility) because they know me, I have grown up from this village so they know I am still young. They will say first get pregnant and you give birth to your child yet for me it is not what I want.” *—*SI user in Uganda, aged 19
Limitations	SI training typically requires face time with a healthcare worker before one can realize the access‐related benefits of SI, an obstacle for those concerned about provider bias or discrimination	“If you go to the hospital and you are underage… like if you have not reached that age of starting to use things like that. Obviously, the doctor will deny you.” —Condom user in Kenya, aged 17	Limitation not mentioned by SI users
	Dispensation of multiple units for use at home may be inconsistent and women may still encounter stockouts when seeking refills (only reported by one SI user)^e^	Limitation not mentioned by those without SI experience	“I made a decision to be using Sayana (DMPA‐SC), only that this time around it is not available, and instead there is Depo only.” *—*SI user in Malawi, aged 28
*Self‐management* benefits	Some women prefer to self‐inject because the overall experience is more comfortable	“If one's hand is heavy by nature it can make an injection pain, but mine is light then I will not feel a lot of pain.” *—*DMPA‐SC user (provider administered) in Uganda, aged 16	“I feel relaxed…Because I am the one doing it to myself.” —SI user in Nigeria, aged 18
	Using SI empowers women to ensure effective and timely self‐administration on their own without depending on the health system	“I can read through and I get to know that this one is expiring this day on this month…but the other one, he or she wants to sell many…so he or she may just bring it (even if expired) and inject me. Me I see that is good to be injecting by myself.” —Withdrawal user in Uganda, aged 34	“The benefit of getting self‐ injection at home is that…you can inject yourself at any time of the day whether during the day or night because you have the Sayana (DMPA‐SC) readily available with you.” *—*SI user in Malawi, aged 28
	Self‐management gives women the opportunity to include partners in cooperative contraceptive use (SI Users in Malawi)^c^	“My husband and I do things together, so he will remind me when I tend to forget that it is time to inject myself and he will also encourage me.” *—*Implant user in Nigeria, aged 36	“My husband asked if he can also be injecting me and I told him that, ‘only if we went together for the training and you also heard the information, that way would also be possible you injecting me.’” *—*SI user in Malawi, aged 40
Limitations	Lack of confidence to manage self‐injection competently	“If you're being injected by the healthcare provider, if there are issues, the healthcare provider will notice but when you're self‐injecting, you might not know what will occur or the symptoms.” —Nonuser of contraception in Nigeria, aged 17	“When I managed to inject myself, I felt so good, even though it was difficult to understand, but I felt good after I injected and it was now clear after injecting myself.” *—*SI user in Malawi, aged 33
	Fear of injections	“Injections are painful, the fear of hurting myself may make me not inject myself properly.” *—*DMPA‐IM user in Uganda, aged 19	“I was scared to inject myself because I had so many worries like what if the needle breaks inside my body what can I do? I had these questions in the first days but now I am used (to it).” *—*SI user in Malawi, aged 31

^a^ Noted only among participants from Malawi

^b^ SI users overcoming privacy limitations was a minor theme as only a few covert users felt uncomfortable storing units at home

^c^ SI users supporting this theme were primarily from Malawi

^d^ This theme was raised by a number of participants with no SI experience in Kenya and Uganda but only referenced by one SI user, potentially because the vast majority of our younger participants using SI were married and doing so with consent from their husbands, therefore less likely experience provider bias

^e^ This was only a minor theme (one SI user reported supply issues with DMPA‐SC); however, it is fundamental to improved access being a legitimate benefit

#### Privacy

The privacy associated with SI was consistently discussed as a benefit of the method across all study countries. Regardless of whether contraceptive use was supported in their particular home context, participants recognized that SI created opportunities to use contraception discreetly in situations where family members disapproved of contraceptive use. Many participants without SI experience pointed to this hypothetical benefit, and, indeed, some SI users confirmed this advantage of SI as they reflected on their own experiences with it. In the words of a 24‐year‐old from Nigeria who was using SI without her husband's knowledge: “I don't feel he will find out when I inject myself; he will not be there and will not know what is going on.”

A second major theme was that the privacy of SI can protect women from community scrutiny in contexts where contraceptive use is considered taboo. For many participants, especially those in Nigeria and Uganda, where stigma was commonly raised as a barrier to contraceptive use, traveling to clinics, waiting in line for an appointment, or just being seen at the health facility posed a risk that their desire to use contraception could be exposed. Avoiding these scenarios by privately self‐injecting offered a path to safer covert use for several SI users in our sample; some participants with no SI experience similarly recognized this advantage of SI. Since sex among adolescents and unmarried women is particularly stigmatized, younger participants especially appreciated a contraceptive option that limited judgment from others. Some nonusers of contraception identified this advantage of SI and implied it lessened the barriers to using contraception, as illustrated in this quote:

It offers the privacy and reduces village gossip. Every time they see you at the health center, in the family planning clinic, they just know that you are doing something to prevent pregnancy, and it becomes common knowledge, but if you can inject yourself from the privacy of your home, then no one will know about it. —Nonuser of contraception in Uganda, aged 18

Related to this theme of privacy from the community, some participants in Malawi (both SI users and those without SI experience) expressed that SI allowed for a shared privacy for one's family in a context where participants primarily described making joint contraceptive decisions with partners. One DMPA intramuscular injection (DMPA‐IM) user, aged 25, explained her interest in SI when stating: “Because it will be only me and my husband who will know that I am on a contraceptive method, not other people.”

Finally, a privacy‐related theme unique to Malawi was SI users appreciated avoiding showing their bodies to providers once able to self‐inject independently. As a 31‐year‐old SI user from Malawi stated: “The other benefit is that it provides privacy to inject yourself and you handle your body alone like you don't need to show your thighs to anyone else.” A few Malawian participants without SI experience could foresee this benefit as well.

*The privacy benefit of SI has limits*. While participants frequently endorsed SI's potential for discreet use, many younger participants, especially those living with parents, highlighted that the privacy of SI was not sufficient when private spaces for injection or storage of units were not readily available. A 17‐year‐old condom user from Kenya stated: “I'm afraid because maybe my mum might come across one and she will have questions to ask me.” Indeed, no adolescent SI users in our sample referenced using SI while hiding their contraceptive use from parents in the home. This may reflect the barrier that insufficient privacy poses for adolescents interested in SI. The adolescent SI users in our sample were less concerned with in‐home privacy needs because they were all either married (typically with consent to use contraception from their partners) or living on their own.

The theme of inadequate privacy was also relevant when interviewees considered the possibility of using SI with a disapproving partner. For example, a 19‐year‐old Ugandan participant using provider‐administered DMPA‐SC suggested that keeping the product at home carried risk for women if they intended to keep contraceptive use hidden from their partners: “If I'm doing it without the consent of my husband, he can easily find it in the house, and it causes problems in our marriage.” In some cases, covert use was described as challenging because side effects such as bleeding changes could reveal contraceptive use; in other cases, the concern was about storage or disposal of doses. Several SI users in our sample described using SI at home without a disapproving partner knowing; among these SI users, a few addressed the issue of limited privacy by choosing to access and self‐administer the injection at the community health outpost or the home of the healthcare provider as opposed to storing units at home.

#### Eased Access Barriers

Across all study contexts and regardless of experience with contraception, participants viewed SI as an option that could make contraception more accessible. Participants identified multiple subthemes that all supported SI's potential to help women execute a decision in accordance with their contraceptive preferences: time savings, avoidance of stockouts, cost savings, and not having to leave home to access contraception. According to many participants, such access improvements enabled contraceptive continuation that would not have been possible with methods requiring regular clinic visits.

First, *time savings* was frequently identified as an advantage by SI users and participants without SI experience. For those who worked long hours or had to travel far to access health services, attending clinics was an opportunity cost. This was an especially common theme among Malawian participants living in rural areas who were accustomed to long treks to health facilities when seeking contraceptive services. Some SI users experienced this advantage as freedom to act according to their contraceptive preferences without sacrificing wage‐earning hours or time tending to other responsibilities, as demonstrated by the following quote from an SI user:

I now manage to do the work that I was not able to do during the time that I was going to the hospital to get family planning methods and I don't have to get worried about going to the hospital because I now have my own clinic in my house. —SI user in Malawi, aged 40

Second, *avoiding stockouts* was identified as a major advantage that facilitated the desired continuation of contraception by SI users and nonusers alike. A 31‐year‐old Malawian SI user described a scenario she had experienced with DMPA‐IM, “Sometimes Depo injection method runs out of stock at the community HSAs place and if your dates are due…you might not be able to access the injection, hence, you may end up getting pregnant.” This was an especially profound advantage for this participant because she had indeed experienced a pregnancy when the DMPA‐IM method she was using was out of stock. As she explained:

This really affected my life because I gave birth to a child I did not plan for. So, when this self‐injection method came, I thought that it is a good method because I cannot experience what I experienced with the last method. —SI user in Malawi, aged 31

While participants across all countries were familiar with the frustration of stockouts and many spoke to this benefit of the method, SI users in Malawi often referenced having a supply for “the whole year” and were, therefore, especially likely to highlight the security they felt in having multiple units at home.

Third, *cost savings* was identified as an advantage that facilitated contraceptive continuation in line with one's preferences by both SI users and others who saw the hypothetical benefit. SI users reported experiencing financial benefits of SI mostly in relation to a reduction in transport costs and disruptions to work. For example, this SI user stated:

You save on transport and you don't miss any work that you were supposed to do. It takes a little time to inject yourself at home compared to the time needed to the health advisor to take other contraceptive methods. —SI user in Malawi, aged 38

Another SI user from Malawi shared she would struggle to get contraception if she regularly needed money for transportation to see a provider. Avoiding provider visit costs was an additional benefit raised by participants in Nigeria (only one SI user) and, to some extent, in Kenya. As a 38‐year‐old oral contraceptive user from Nigeria stated: **“**When you inject yourself, it is free, but if another person [a provider] injects you, it will cost money.”

A fourth theme was being able to access contraception even *when leaving home was not possible*. For example, some participants mentioned needing to stay home to care for a child or an ill relative, and the option to self‐inject was a meaningful advantage of the method. An SI user in Malawi experienced this benefit firsthand when she was unable to leave her home and access health services due to COVID‐19:

When this pandemic was at peak, I already had the injectable contraceptive method at my home for the whole year, so to me, I wasn^’^t affected with this situation. Even though there were restrictions, I still had my injections and I will still have my injections with or without COVID‐19. —SI user in Malawi, aged 31

An additional Nigeria‐specific example under this theme was that SI could facilitate desired contraceptive continuation during episodes of community insecurity that might impede their ability to safely travel to clinics.

Finally, a theme in Kenya and Uganda was that access‐related advantages of SI allow women (particularly adolescents) to avoid anticipated provider bias, mistreatment, and/or scrutiny when continuing contraceptive use. Adolescent participants without SI experience in Kenya and Uganda described this hypothetical advantage; for example, a participant intrigued by the benefits of SI reflected on her past experiences with health providers:

Another benefit is that I will not go there [to the clinic] to be asked questions: “Why are you using?”…“How many partners do you have?,” “Why do you want [contraception]?”… “How many children do you want?”…Such questions. —Condom user in Kenya, aged 19

This theme was not common in the experiences of adolescent SI users in our sample who were primarily married and parous; one exception was a nulliparous teen living without her parents in Uganda who spoke of avoiding health facilities because she felt pressure from providers to have a child before starting contraception. This participant accessed SI training and DMPA‐SC units from a community health worker and appreciated not having to engage with providers for reinjection.

*Easier access is sometimes not easy enough*. Several participants called attention to the *limitations* of SI's potential to ease access barriers when they discussed general challenges to contraceptive access that they saw as extending to SI. This was most common among young participants, especially nonusers of contraception or condom users, who often expected requests for contraception to be rejected by providers or assumed they would be “judged.” These expectations tempered interest in SI among many adolescent participants. A Kenyan participant who had never used contraception expressed doubt that she could get the injection without her mother present, thus negating the privacy benefit of SI:

The clinician might be surprised because of my age; I am quite young. He might even refuse, maybe if my mother accompanies me to the facility and explains to them, I think after the explanation I will be able to get the injection. —Nonuser of contraception in Kenya, aged 16

Additionally, it is important to recognize that the benefits of having a supply at home are contingent upon consistent stock availability (a continual challenge in many places; Zuniga et al. 2022) and providers’ willingness to dispense multiple units to women for home use. Issues related to provider stock of DMPA‐SC were not a prominent theme in data from SI users in our study. However, one SI user in Malawi did report needing to use provider‐administered DMPA‐IM when DMPA‐SC was unavailable.

#### Self‐Management

Self‐management encompasses not only the various steps to self‐administer DMPA‐SC but also managing the timing of injections, supply and disposal, and understanding problematic side effects to ensure safe and effective use. A major theme from interviews with SI users, most of whom were familiar with provider‐administered injectable methods, was that SI was a more optimal mode of administration. SI users commonly described that having the opportunity to be “calm and gentle” with their own body and/or administer the contraceptive in a comfortable setting made for a more “peaceful” experience compared to injectable methods that are provider‐administered. Even some participants who had never self‐injected were able to appreciate this potential advantage of SI; as a 35‐year‐old condom user from Nigeria stated: “If I can be able to handle it on my own, I prefer to do it for myself at home. You don't stress yourself…You do your thing at the comfort of your home.” Many SI users across study sites also reported that having the unit in their own hands decreased their anxiety around injections. For some, the experience of anticipating an injection coming from someone else caused them to fear being injected, and managing the injection on their own was less stressful. For example, an SI user from Malawi stated:

Some doctors make you feel a lot of pain when injecting, but when injecting yourself you feel good, even if you have to feel pain when injecting yourself you know that you are doing it on your own, so the pain feels different compared to when someone is injecting. —SI user in Malawi, aged 33

Another major theme among users of SI, as well as those with no SI experience, was that self‐management allows one to *ensure effective and timely contraceptive use* without depending on the health system. Particularly nonadolescent participants welcomed the idea of not having to involve others in scheduling, supply management, and verification of expiration dates, as displayed in the following quote:

It will be easier because you'll do it by yourself, you confirm the expiry date, you know everything because you are the person who is having it before you to use it. Compared to pharmacy, maybe someone is bringing something which had already expired because that person is just after money…So this will be easier because you check the expiry by yourself, you know, and you inject it. —DMPA‐IM user in Kenya, aged 34

Those without SI experience appreciated the “freedom” to re‐inject in a timeframe aligned with their schedule. A 20‐year‐old DMPA‐IM user from Kenya stated, “Maybe someone is at work and they are not able to get time…but when you have it with you, you will be able to just administer it and it will be okay.” SI users noted that indeed through self‐management the opportunity to control when to re‐inject within the appropriate dosing schedule allowed them to navigate their days better and minimize risk of scheduling conflicts which could lead to a missed dose or even an unplanned pregnancy. As a 17‐year‐old SI user from Uganda stated, “I feel in control, because I inject myself at my own time, in my own house when I want.” Some participants noted that in contexts where family members were unsupportive, control over the exact timing of SI could make covert use more feasible as compared to methods that require a provider–client touchpoint for refill/re‐injection. Though this benefit was not directly articulated by SI users in our sample who were using without partner knowledge, others identified the value of timing SI to occur when disapproving family members were not present:

Let us say you're home alone, say your husband is on safari and he is coming back on the weekend then you say let me inject myself on Thursday or Friday. So also that gives you peace because you can inject yourself whenever you want. —Nonuser of contraception in Uganda, aged 20

Some participants noted that SI could give women who valued *partner involvement* in contraceptive use an opportunity to invite partners into their experience in a way that was satisfying; this theme was most prominent in Malawi. Several participants with no SI experience highlighted this as a potential benefit to them and their relationship; SI users in Malawi echoed this theme with reports of their lived experience using cooperatively with partners. A 28‐year‐old SI user in Malawi described the value of this form of support: “My husband injects me, and I feel that this is good, and it promotes bondage and love with my husband.”

Finally, a noteworthy theme specific to SI users’ experiences was that self‐injecting led to increased self‐assuredness over time. After being trained and successfully self‐injecting, in many cases, SI users expressed a boost in confidence related to doing “the job that health workers do”. A 17‐year‐old SI user in Uganda stated, “It has built my capacity, and I am now more confident since I can now inject myself, something that I didn't think that I could do before.” While we did not classify this as a benefit of the method itself, it was a positive byproduct of using it. This “capacity building” was most pronounced among participants from Malawi and Uganda, and even seen among some who felt they had no control over contraceptive decisions themselves. Several participants spoke with enthusiasm about their evolution from being afraid to self‐inject to competently doing so; this transformation inspired a desire to engage with other women and support them through the decision process and learning to feel comfortable with SI. As an adolescent SI user in Uganda stated, “In future, I want to support other people who may be afraid of injecting themselves…or even teach them to inject themselves.”

*Fear prevails among those who have never tried SI*. While the benefits of *self‐management* were appreciated by some participants who had never used SI, they were more universally cited among SI users. In fact, among participants with no SI experience, it was common to have trepidation around self‐management; these participants raised an array of concerns related to low self‐efficacy to self‐inject. They feared injecting incorrectly or at the wrong time and believed such mistakes could render the method ineffective, result in unplanned pregnancy, or cause an overdose. Adjacent to this fear, some participants were concerned that SI meant they would be managing their contraceptive experience entirely on their own without the help of a provider or family member. Thus, they feared being unable to manage side effects or unexpected reactions to the injection as well as having to remember when to use the method without reminders from others. For example, a 44‐year‐old condom user from Nigeria stated: “There may be a problem and you can wound yourself. You cannot run to anybody because you did it by yourself.”

Needle‐specific fears were also common across study sites; participants worried about the pain of the injection or that the needle could break inside their bodies. Many participants noted that SI was best suited for the “bold” or “courageous.” A 20‐year‐old DMPA‐IM user from Kenya stated: “I feel that I should be brave to be able to inject myself because injection is very painful, so lack of courage to overcome the fear of the pain might make one not use it.”

## DISCUSSION

We identified specific ways in which the self‐reported benefits of SI hold the potential for it to live up to the promise of giving women greater control over contraceptive use (Askew and Wells 2018; PATH, n.d.). Our findings from a diverse group of women (some of whom had used SI and some who had not) across four sub‐Saharan African countries suggest ways in which SI is uniquely beneficial compared to other contraceptive methods. We identified major themes related to SI offering *privacy* (compared to methods that risk visibility—either of the user or the method), *easier access* (compared to methods that require clinic visits), and *self‐management* (compared to methods that require provider administration).

Related to *privacy*, our findings confirmed that women themselves see value in the potential of SI to give women control over who knows about their contraceptive use, either at home or in the community, making it easier to use for those with disapproving or controlling family members. Indeed, SI users in our sample spoke of SI helping to keep their “secret safe” from a partner or, more commonly, from in‐laws or others in their community outside of their household. Participants familiar with DMPA‐IM felt that avoiding periodic provider visits where they were likely to be seen was a specific advantage of SI. Related to *easier access*, participants articulated a plethora of ways in which SI could facilitate action in line with a decision to use contraception through improved access. SI users described time savings, cost savings, and the ability to avoid stockouts and physical access barriers as advantages; others who had not tried SI echoed these as hypothetical benefits. Many participants expressed frustration with past experiences with DMPA‐IM stockouts; while supply issues can and will occur with DMPA‐SC, the idea of safeguarding one's own, albeit small, supply at home was reassuring to many women. Adolescents in Uganda and Kenya further pointed out that the option to self‐inject could hypothetically help them avoid anticipated provider bias, mistreatment, and/or scrutiny when continuing contraceptive use. Finally, related to *self‐management*, SI users described the benefit of injecting oneself as allowing for a more comfortable and confidence‐building experience, and SI users and nonusers alike commonly appreciated that SI could better ensure more effective and timely contraceptive use compared to provider‐administered methods.

These findings speak to multiple ways in which SI may live up to its “empowering” (Askew and Wells 2018; PATH, n.d.) potential by helping some women overcome social and structural barriers to using contraception. Yet, while women perceived benefits of SI, they also articulated barriers to realizing these benefits—particularly for those whose agency and autonomy are most constrained. Unmarried adolescents, especially those living with parents, often cited concerns about privacy and managing self‐care. Adolescents often struggle to be assertive with sexual and reproductive health decision‐making, especially in repressive environments (Ahinkorah et al. 2020); this tendency towards lower self‐efficacy may make them less likely to try SI. Lower desire to initiate SI—even when viewed favorably— among adolescents as compared to women of all reproductive ages bears out in the literature on SI feasibility and acceptability (Burke, Packer, et al. 2018; Cover, Ba, et al. 2017; Cover, Lim, et al. 2017). Women with partners opposing contraceptive use may also find it difficult to realize the benefits of SI. Though some older covert users in our sample were adeptly using SI, for other participants with disapproving partners, the benefits of SI were not appreciable enough to outweigh their concerns about being discovered or the power dynamics of their relationship. Women needing to use contraception covertly may more acutely experience apprehension about managing self‐care given the low social support that presents challenges for them at baseline (Kibira et al. 2020). Finally, fear was the most cited barrier to SI and was universal across contexts and ages. While many SI users reflected favorably on their evolution from questioning their capacity to self‐inject to embracing their new confidence, with some even wanting to teach and support other women interested in SI, initial fears appear to play a major role in low rates of SI uptake across study sites.

While our results support and give nuance to the potential of SI to give women more control of contraceptive use *in some cases*, they also clearly show that the technological fix of SI has, unsurprisingly, not fully addressed structural barriers like inequitable gender norms that pose barriers to women's ability to use contraception when they want to. Despite the reality of the need for more profound social change to fully enable women to have agency and autonomy related to contraception, our findings point to several shorter‐term SI programmatic innovations with the potential to optimize further scale up. First, related to access barriers, solutions that reduce the need for women to go to a health provider for training or to obtain DMPA‐SC units are greatly needed. Training videos, such as the one developed by John Snow Inc. and PATH (Access Collaborative, n.d.‐a) that allow women to receive initial or refresher training at home could bolster women's ability to use SI discreetly. A digital training platform for SI via WhatsApp coupled with the delivery of the first unit of DMPA‐SC by motorcycle courier in Kenya is a promising example of solutions needed for those who find the initial in‐person training to be a barrier to SI use (Mills et al. 2023). Finally, while challenges related to inadequate supply of DMPA‐SC were not a major theme in our data, supply chain management, and consistent dispensation practices are critical to women taking full advantage of the access‐related benefits of SI.

Further, programmatic solutions are needed to address women's fear of SI. It is well established that women need contraceptive counseling that instills confidence and helps them understand that self‐care options can include the support of a healthcare worker if desired (World Health Organization, n.d.). Bolstering self‐efficacy for women and educating them about channels of support may reduce the prohibitive fears some currently express about SI. A study in Malawi tested SI counseling verbiage that addressed user‐specific concerns and found it led to an uptick in SI use until supply chain issues interfered (Burke, Packer, et al. 2022). Peer support programs have shown promise to complement provider counseling. ICAN designed and evaluated community‐based programs in Uganda and Malawi that utilize peer mentors to educate and support fellow women with contraceptive decision‐making and (among interested women) with SI (results forthcoming). Partners offer another potential source of support for women interested in SI. A 2022 study on men's role in DMPA‐SC SI showed men in Malawi can and are helping their partners cooperatively use SI through direct hands‐on assistance and emotional support to reduce fears and improve self‐efficacy (Ruderman et al. 2022); these themes are substantiated by our findings in Malawi. As the article notes, this path should be explored in cases where women desire partner involvement and it can augment a woman's self‐confidence as she uses SI, not detract from it. In summary, a more holistic approach to counseling and support that gives attention to women's concerns and hesitations around SI may yield more empowered contraceptive decisions as women consider all of their options, whether they choose SI, another method, or not to use contraception at all.

Finally, it is important to note that all the perceived benefits of SI identified by women in our study were related to helping individuals *act* on the decision to use contraception rather than improving their ability to *make* an empowered decision for oneself about whether or not to use contraception. This is not surprising given the inherent limitations in what a new contraceptive device can do to empower people but is important to mention as a reminder of the need to couple the roll‐out of new contraceptive technologies with programs that aim to not only give women access to a new tool to control their fertility but also support them in making empowered decisions about whether or not to use contraception in the first place.

### Limitations

A strength of this study is that we interviewed a diverse group of women purposively sampled to represent a range of contraceptive experiences, including those with a history of SI use. We captured a wide spectrum of perspectives on the method's benefits and influence on a woman's contraceptive experience. Additionally, the diversity of contraceptive experiences in our sample allowed us to juxtapose the perceived benefits and challenges of using SI against the realized benefits that SI users reported. Nonetheless, we note several limitations to the study. First, the relatively small number of SI users within each country's sample (and the lack of any SI users in the Kenya sample due to extremely low use rates in the country during recruitment) limited our ability to explore differences in SI use experiences by country and by sociodemographic characteristics to the extent we were able to with the larger non‐SI user sample. Second, differences in sampling between countries posed a challenge for drawing conclusions about how themes differed across settings. Data from Kenya and Nigeria skewed urban, while the Malawian participants were almost all rural residents. Since urban residents tend to have better healthcare access and information than their rural counterparts, these differences limited our ability to draw comparisons across countries, although we noted in the text when we felt these differences affected our analysis. Additionally, participants were recruited from the private pharmacy sector in Kenya and Nigeria but not Malawi or Uganda, which may have influenced perceptions of SI cost, access, or feasibility. However, cost was not a challenge highlighted by our sample.

## CONCLUSION

We identified unique benefits and challenges to using SI through interviews with a diverse group of women in four countries in sub‐Saharan Africa. The benefits of SI—privacy, eased access barriers, and self‐management—manifested in different ways for different women but collectively point to SI's potential to augment women's sense of control over their contraceptive use in certain cases. At the same time, our findings shed light on potential reasons for the low uptake of SI across sub‐Saharan Africa as they highlight the prevalent fear women have of injecting themselves and how the technological advancement of SI does not fully address inequitable gender norms and other structural barriers that constrain women's ability to use any form of contraception, including SI when they want to. Programmatic solutions that specifically target these barriers by increasing women's self‐efficacy to self‐inject, if they so choose, and making training and method procurement easier and more discreet may further optimize the unique potential of SI.

## AUTHOR CONTRIBUTIONS

EH performed analysis, conceptualization, visualization, and writing of the original draft. LS, CB, SG, SO, GN, ZK, PaW, LN, LA, MN, CO, AT, JV, AJ, and SD performed project administration, analysis, writing review, and editing. AM, PA, BK, AK, TJ, and IM performed project administration, analysis, and writing reviews. SC and BP performed project administration, writing review, and editing. MK, EO, JL, DA, EB, AdM, PeW, and KH performed study conceptualization and writing review with MK, EO, JL, DA, and KH participating in editing as well.

## FUNDING INFORMATION

This research was supported by the Bill & Melinda Gates Foundation under Grant number OPP1216593.

## CONFLICT OF INTEREST STATEMENT

We have no conflicts of interest to disclose.

## ETHICS STATEMENT

Ethical approval for the research was obtained from: the UCSF Institutional Review Board (270555, 270747, 270554, 270084); the Kenya Medical Research Institute's Scientific Ethics Review Unit (KEMRI/SERU/CMR/P00136/4013), and Kenyan National Commission for Science, Technology and Innovation (464643); the Makerere University School of Public Health Research and Ethics Committee (812) and Uganda National Council of Science and Technology (HS1087ES); the Malawi University of Science and Technology Research Ethics Committee (P.03/2020/007); and the National Health Research Ethics Committee, Nigeria (01/01/2007‐25/09/2020).

## Data Availability

The qualitative data in this study are jointly owned by the ICAN research consortium partners and not publicly available due to the need to ensure a high degree of respondent confidentiality consistent with our informed consent process. Requests for data can be made through the corresponding author and will be considered on a case‐by‐case basis. Additionally, the authors can make their data collection instruments (interview guides) and analysis tools (codebook for Dedoose qualitative data analysis software) available.
